# Leucine‐rich *α*‐2 glycoprotein promotes lung fibrosis by modulating TGF‐*β* signaling in fibroblasts

**DOI:** 10.14814/phy2.13556

**Published:** 2017-12-26

**Authors:** Hiromi Honda, Minoru Fujimoto, Satoshi Serada, Hayato Urushima, Takashi Mishima, Hyun Lee, Tomoharu Ohkawara, Nobuoki Kohno, Noboru Hattori, Akihito Yokoyama, Tetsuji Naka

**Affiliations:** ^1^ Center for Intractable Immune Disease Kochi Medical School Kochi University Nankoku Japan; ^2^ Laboratory of Immune Signal National Institutes of Biomedical Innovation, Health and Nutrition Ibaraki Japan; ^3^ Department of Anatomy and Regenerative Biology Osaka City University Graduate School of Medicine Osaka City University Osaka Japan; ^4^ Hiroshima Cosmopolitan University Hiroshima Japan; ^5^ Department of Molecular and Internal Medicine Graduate School of Biomedical Science Hiroshima University Minami‐ku, Hiroshima Japan; ^6^ Department of Haematology and Respiratory Medicine Kochi Medical School Kochi University Nankoku Japan

**Keywords:** Fibroblast, Leucine‐rich alpha‐2 glycoprotein, pulmonary fibrosis, Smad2, transforming growth factor‐beta

## Abstract

TGF‐*β* has an important role in fibrotic diseases, including idiopathic pulmonary fibrosis (IPF). Detailed analysis of TGF‐*β* signaling in pulmonary fibrosis at the molecular level is needed to identify novel therapeutic targets. Recently, leucine‐rich alpha‐2 glycoprotein (LRG) was reported to function as a modulator of TGF‐*β* signaling in angiogenesis and tumor progression. However, the involvement of LRG in fibrotic disorders, including IPF, has not yet been investigated. In this study, we investigated the role of LRG in fibrosis by analyzing LRG knockout (KO) mice with bleomycin‐induced lung fibrosis, an animal model of pulmonary fibrosis. The amount of LRG in the lungs of wild‐type (WT) mice was increased by bleomycin administration prior to fibrosis development. In LRG KO mice, lung fibrosis was significantly suppressed, as indicated by attenuated Masson's trichrome staining and lower collagen content than those in WT mice. Moreover, in the lungs of LRG KO mice, phosphorylation of Smad2 was reduced and expression of *α*‐SMA was decreased relative to those in WT mice. In vitro experiments indicated that LRG enhanced the TGF‐*β*‐induced phosphorylation of Smad2 and the expression of *Serpine1* and *Acta2*, the downstream of Smad2, in fibroblasts. Although endoglin, an accessory TGF‐*β* receptor, is essential for LRG to promote TGF‐*β* signaling in endothelial cells during angiogenesis, we found that endoglin did not contribute to the ability of LRG to enhance Smad2 phosphorylation in fibroblasts. Taken together, our data suggest that LRG promotes lung fibrosis by modulating TGF‐*β*‐induced Smad2 phosphorylation and activating profibrotic responses in fibroblasts.

## Introduction

Idiopathic pulmonary fibrosis (IPF) is a chronic progressive disease with a poor prognosis (Raghu et al. 2011, 2015). The pathogenesis of the disease is considered to be abnormal wound healing following lung injury and is characterized by proliferation of fibroblasts and deposition of extracellular matrix (ECM) (Rafii et al. 2013). Two antifibrotic drugs that target fibrotic response are clinically used at present. Pirfenidone inhibits production of growth factors such as transforming growth factor (TGF)‐*β*, basic fibroblast growth factor (bFGF), and platelet‐derived growth factor (PDGF) (Schaefer et al. 2011). Nintedanib is a small molecule that inhibits the activity of tyrosine kinase receptors, such as vascular endothelial growth factor (VEGF) receptor, PDGF receptor, and FGF receptor (Wollin et al. 2014). Although there is evidence that these drugs successfully slow the decline in forced vital capacity over a year (Fleetwood et al. 2017), the therapeutic responses differ between individuals (Loeh et al. 2015), and the effectiveness of antifibrotic therapies remain limited. Therefore, it would be useful to elucidate the molecular mechanism of fibrosis in IPF to identify new potential targets for treatment of this disease.

Bleomycin‐induced lung fibrosis in mice is widely studied animal model of pulmonary fibrosis (Walters and Kleeberger 2008). Although this model does not sufficiently recapitulate the pathogenesis of human IPF, research using this model has provided important insights into the pathology of lung fibrosis including IPF (Moore and Hogaboam 2008; Peng et al. 2013; Bauer et al. 2015). The roles of TGF‐*β* in the fibrosis process in the lungs are well documented in humans and mouse models (Zhang et al. 1996; Cutroneo et al. 2007; Gu et al. 2007; Xia et al. 2008). In the bleomycin‐induced lung fibrosis model, TGF‐*β*‐induced Smad2 activation in the lung has been found to be the major pathway in fibroblast activation (Dong et al. 2015), differentiation, and ECM production (Higashiyama et al. 2007; Koh et al. 2015). Thus, understanding the regulation of TGF‐*β*‐Smad signaling in mouse models of lung fibrosis will help to elucidate the pathogenesis of pulmonary fibrosis.

Several therapeutics targeting TGF‐*β* have been developed for fibrotic diseases (Aschner and Downey 2016). However, considering that TGF‐*β* has homeostatic and physiological functions, such as regulation of inflammation and vascular development, the global or complete inhibition of TGF‐*β* might have undesired side effects. Therefore, instead of completely inhibiting TGF‐*β* function, a therapeutic approach that targets TGF‐*β* only at sites of progressive fibrosis is desirable. For example, *α*v*β*6 integrin, which is required for the activation of latent TGF‐*β*, is upregulated specifically at sites of lesions in the lung (Munger et al. 1999). Targeting of *α*v*β*6 integrin leads to local inhibition of TGF‐*β* activation in IPF lungs, and a clinical trial using monoclonal antibody against *α*v*β*6 integrin is currently underway (NCT01371305) (Horan et al. 2008).

We have previously reported that serum leucine‐rich alpha 2‐glycoprotein (LRG) levels are elevated in chronic inflammatory diseases, such as rheumatoid arthritis and ulcerative colitis (Serada et al. 2010, 2012; Fujimoto et al. 2015). In addition, studies using mouse models have indicated that LRG increases in inflammatory diseases, such as collagen‐induce arthritis and dextran sulfate sodium‐induced colitis (Serada et al. 2012; Urushima et al. 2017), implying that evidence from animal studies will help us to understand the role of LRG in human diseases. LRG is a member of the leucine‐rich repeat family and contains eight repeating consensus sequences, each of which consists of 24 amino acid residues (Haupt and Baudner 1977; Takahashi et al. 1985). Recently, LRG was reported to interact with TGF‐*β* or receptor complexes and modulate TGF‐*β* signaling. In endothelial cells, LRG has been reported to interact with the TGF‐*β* receptor complex, including endoglin, and promote TGF‐*β* signaling by activating the ALK1‐Smad1/5 pathway (Wang et al. 2013). In addition, we have reported that LRG enhances TGF‐*β*‐Smad2 signaling in several cell types, including cancer cells in a xenograft model and T cells in an experimental arthritis model (Takemoto et al. 2015; Urushima et al. 2017). To understand the involvement of LRG in IPF, we investigated the effect of LRG on fibrotic processes in an animal model of pulmonary fibrosis using LRG KO mice. Our results suggest that LRG enhances the TGF‐*β*‐induced Smad2 phosphorylation in fibroblasts and promotes fibrosis.

## Materials and Methods

### Mice

C57BL/6 wild‐type (WT) mice were purchased from Jackson Laboratory (Bar Harbor, Maine, USA) and LRG knockout (LRG KO) mice on a C57BL/6 background were generated as previously described (Takemoto et al. 2015). WT and LRG KO mice were maintained under specific pathogen‐free conditions at the National Institutes of Biomedical Innovation, Health and Nutrition (NIBIOHN, Osaka, Japan). All animal experiments were approved by the Institutional Animal Care and Use Committees of NIBIOHN.

### Bleomycin‐induced lung fibrosis model

To induce lung fibrosis, 1.5 mg/kg of bleomycin (Nippon Kayaku Co., Tokyo, Japan) was intratracheally administered to 8–12‐week‐old female mice as described previously (Senoo et al. 2010). Briefly, mice were anesthetized with an intraperitoneal injection of 50 mg/kg sodium pentobarbital (Kyoritsu Seiyaku, Tokyo, Japan), and the trachea was exposed by a cervical incision. Bleomycin was dissolved in phosphate‐buffered saline (PBS) and intratracheally instilled using a 30‐gage needle. Mice were sacrificed at different time points for histological and biochemical analysis. Control animals were sacrificed without treatment.

### Bronchial alveolar lavage fluid (BALF) collection and enzyme‐linked immunosorbent assay (ELISA)

Mice were sacrificed on days 1, 7, 14, and 21 after bleomycin treatment, and the trachea was cannulated with a 20‐gage indwelling needle. Lungs were lavaged three times with 0.5 mL of PBS for each lavage. The lavage fluids were centrifuged at 400*g* for 5 min at 4°C, and the supernatants were stored at −80°C until the measurement of LRG and TGF‐*β*1 concentrations. Total cells in BALF were counted using a hemocytometer. Protein concentration in the supernatant of BALF was measured using a DC Protein Assay Kit (Bio‐Rad Laboratories, Hercules, CA, USA). Concentrations of LRG and TGF‐*β*1 were measured by ELISA. For the measurement of LRG, monoclonal antibodies mLRA0010 and rLRA0094 were used as previously described (Honda et al. 2016). The specificity of these antibodies was confirmed using the BALF and serum from a LRG KO mouse. The concentrations of BALF TGF‐*β*1 were measured according to the manufacturer's protocols using the TGF‐*β*1 ELISA kit (eBioscience, San Diego, CA).

### Histology

At the time of sacrifice, the lungs were inflated with 4% paraformaldehyde (PFA)–PBS at a pressure of 25‐cm H_2_O. The trachea, hearts, and lungs were dissected en bloc and further fixed with 4% PFA–PBS for 24 h. Fixed tissues were dehydrated and embedded in paraffin. Paraffin sections of 4‐μm thickness were prepared and used for H&E staining, Masson's trichrome staining, or immunohistochemical analyses.

### Immunohistochemistry

Sections were stained with anti‐LRG (IBL, Gunma, Japan) and anti *α*‐SMA (abcam, Cambridge, UK) antibodies. Primary antibodies were visualized using an Avidin‐Biotin Complex detection system (Vector Laboratories Inc., Burlingame, CA, USA).

### Hydroxyproline assay

Collagen deposition was assessed by quantifying the hydroxyproline content in whole lung tissues as described previously (Woessner 1961; Hattori et al. 2000) with modifications. At the time of sacrifice, both lungs were harvested and hydrolyzed in acidic condition. Samples were then mixed with citrate–acetate buffer. Chloramine‐T solution was added, and the mixture was incubated for 30 min at room temperature. Ehrlich's solution was added, and the samples were incubated further at 65°C for 30 min. Absorbance was measured at 550 nm, and the amount of hydroxyproline (*μ*g) contained in whole lung was calculated.

### Cell culture

Mouse fibroblast cell lines (L929 and NIH/3T3) and a human lung cancer cell line (A549) were obtained from the Japanese Collection of Research Bioresources Cell Bank (Osaka, Japan). Mouse fibroblasts and A549 were maintained in Dulbecco's Modified Eagle's Medium or RPMI1640 medium, respectively, supplemented with 10% fetal bovine serum (Serum Source International, NC, USA), 100 U/mL penicillin, and 100 *μ*g/mL streptomycin (Nacalai Tesque, Kyoto, Japan).

### Generation of mouse LRG‐transfected cell lines

To obtain cell lines that stably express mouse LRG, L929 cells were transfected with the pcDNA3.1‐Lrg1‐V5/His expression vector as described previously (Kim et al. 2009; Takemoto et al. 2015). Cells were selected in media containing 500 *μ*g/ml of Geneticin (Invitrogen, Carlsbad, CA, USA), and the clone that expressed LRG were used for further experiments (pcDNA‐Lrg1, clone L15). Control cell lines were also established by stable transfection with an empty vector (pcDNA, clone E2).

### Purification of recombinant mouse LRG

A549 cells were transfected with pEBMulti‐Neo‐Lrg1 vector (Wako, Osaka, Japan) to obtain mouse LRG‐expressing cells. Cells were cultured for 72 h in serum‐free RPMI 1640 medium (Wako). LRG secreted into culture supernatants was purified using an antibody affinity column (NHS‐activated Sepharose 4 Fast Flow conjugated with anti‐mLRG antibody mLRA0010) and concentrated by ultrafiltration (Amicon Ultra 10K; Merck Millipore, Darmstadt, Germany). The concentration of LRG was determined by performing mouse LRG ELISA described above.

### Western blot analysis

Protein levels of LRG, phospho‐Smad2, phospho‐Smad1/5/8, Smad2, endoglin, Id1, and glyceraldehyde‐3‐phosphate dehydrogenase (GAPDH) in lung extracts and cell lysates were analyzed using western blot analysis. Briefly, protein lysates were prepared from lung tissues and cultured cells in radioimmunoprecipitation buffer [10 mmol/L Tris–HCl (pH 7.5), 150 mmol/L NaCl, 1% (v/v) NP‐40, 0.1% (w/v) sodium dodecyl sulfate (SDS), 0.5% (w/v) sodium deoxycholate] supplemented with 1% protease‐inhibitor and phosphatase‐inhibitor cocktails (Nacalai Tesque, Kyoto, Japan). Samples were separated by SDS‐polyacrylamide gel electrophoresis for membrane blotting. The primary antibodies used for western blotting were anti‐phospho‐Smad2 (Ser465/467), anti‐Smad2, anti‐phospho‐Smad1/5 (Ser463/465)/8 (Ser426/428) (Cell Signaling Technology, Beverly, MA, USA), anti‐GAPDH (Santa Cruz), anti‐mouse Endoglin, anti‐Id1 (Santa Cruz), and anti‐mouse LRG (IBL). The experiment was performed at least in triplicate and the bands were quantified by densitometry using ImageJ software (ImageJ 1.51j8, National Institutes of Health, Bethesda, MD, USA). The representative blots and results from densitometric analysis are shown in figures.

### Quantitative PCR analysis

L929 cell samples were prepared in triplicate for PCR analysis. Total RNA was isolated from cells and reverse‐transcribed using the RNeasy Mini and QuantiTect Reverse Transcription Kits (QIAGEN, Tokyo, Japan), respectively. Real‐time quantitative polymerase chain reaction (qPCR) was performed on an ABI PRISM 7900HT Real‐time system (Applied Biosystems, Darmstadt, Germany) using SYBR Premix Ex Taq (Takara Bio, Shiga, Japan). Target gene expression levels were normalized to hypoxanthine phosphoribosyl transferase 1 (*Hprt1*) levels for each sample. The primers for qPCR were designed and used as follows: *Lrg1* (NM_029796), sense 5′‐GGAGCAGCTATGGTCTCTTG‐3′, antisense 5′‐AGTATCAGGCATTCCTTGAG‐3′; *Serpine1* (M33960), sense 5′‐TCTTGCATCGCCTGCCAT‐3′, antisense 5′‐GGACCTTGAGATAGGACAGTGCTT‐3′; *Id1* (NM_010495), sense 5′‐AGCCCTTCAGGAGGCAAGAG‐3′, antisense 5′‐GCGGTAGTGTCTTTCCCAGAGAT‐3′; *Acta2* (NM_007392), sense 5′‐TCTCTATGCTAACAACGTCCTGTCA‐3′, antisense 5′‐CCACCGATCCAGACAGAGTACTT‐3′; and *Hprt1* (NM_013556), sense 5′‐TCAGTCAACGGGGGACATAA‐3′, antisense 5′‐GGGGCTGTACTGCTTAACCAG‐3′.

### siRNA transfection

L929 cells were transfected with mouse endoglin siRNA (Stealth m_siRNA_868; Invitrogen) or non‐targeting siRNA using Lipofectamine reagent (Invitrogen) according to the manufacturer's instruction. Next, 24 h after siRNA transfection, cells were serum‐starved for 24 h before TGF‐*β*1 stimulation.

### Statistical analysis

Data are indicated as means ± standard deviations (SDs). Statistical comparisons between two groups were evaluated using unpaired Student's *t*‐tests. For multiple comparisons, the results were analyzed by ANOVA followed by Tukey's post hoc test. Statistical significance was set at *P* < 0.05. Data analysis was performed using Excel Statistics 2010 (SSRI, Tokyo, Japan).

## Results

### The amount of LRG in the lung was increased by bleomycin administration prior to the development of fibrosis

**Figure 1:**
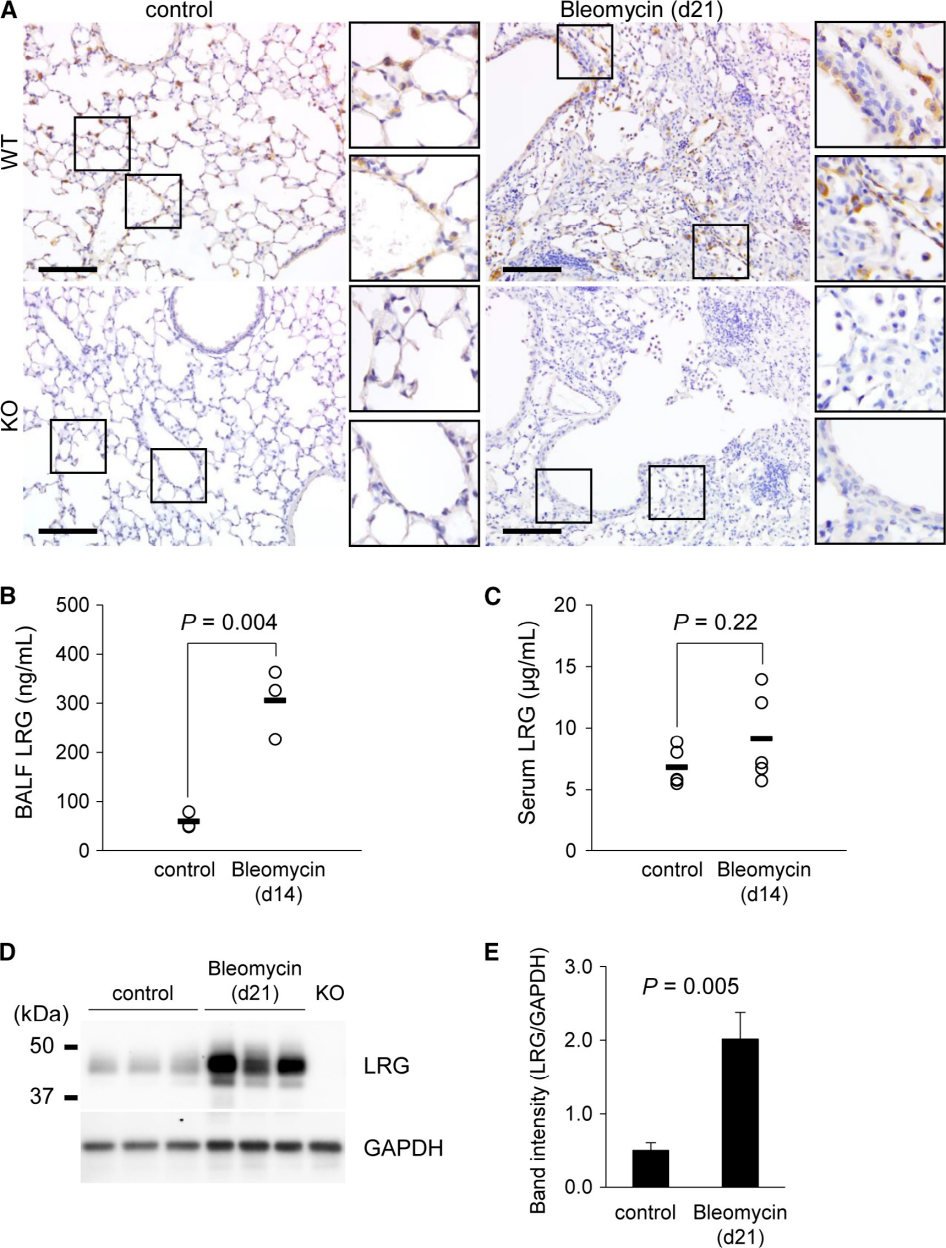
Change in the lung LRG level caused by bleomycin administration. (A) Immunohistochemical analysis of LRG in the control and bleomycin‐administered lung at day 21 from WT and KO mice. Scale bar = 100 *μ*m. (B and C) Concentrations of LRG in BALF (B; *n* = 3) and sera (C; *n* = 5) of WT mice 14 days after bleomycin administration measured by ELISA. The bars (▬) show mean values (B: control: 60.0 ng/mL, Bleomycin: 305.8 ng/mL; C: control: 6.8 *μ*g/mL, Bleomycin: 9.1 *μ*g/mL). (D) Western blot analysis of LRG in the lungs from control and 21 days after bleomycin‐administered mice. GAPDH was used as an internal control. The protein extract from a LRG KO mouse lung was used as a negative control. (E) Quantification of LRG band intensity normalized to GAPDH (*n* = 3). Data were shown as mean ± SD.

To determine whether LRG is implicated in the bleomycin‐induced lung fibrosis model, we examined the localization of LRG in the lung and the concentration of LRG in BALF and serum. Immunohistochemistry of LRG in untreated WT mouse lungs revealed that LRG was expressed in alveolar epithelial cells and endothelial cells. In the lungs collected from WT mice 21 days after bleomycin administration, LRG was intensely stained in infiltrated immune cells and bronchial epithelial cells in addition to alveolar epithelial cells and endothelial cells. The antibody specificity for LRG was confirmed using untreated and bleomycin‐treated KO mouse lungs (Fig. 1A). ELISA analysis showed that the concentration of LRG in BALF (Fig. 1B), but not in sera (Fig. 1C), significantly increased 14 days after bleomycin treatment relative to that in untreated mice. Western blot analysis showed a significant increase in LRG in the lung homogenates 21 days after bleomycin administration (Fig. 1D and E). These results collectively indicate that LRG is increased in many cell types at the lung lesion and may have a role in the pathogenesis of lung fibrosis.

### Bleomycin‐induced fibrosis was suppressed in LRG KO mice

**Figure 2:**
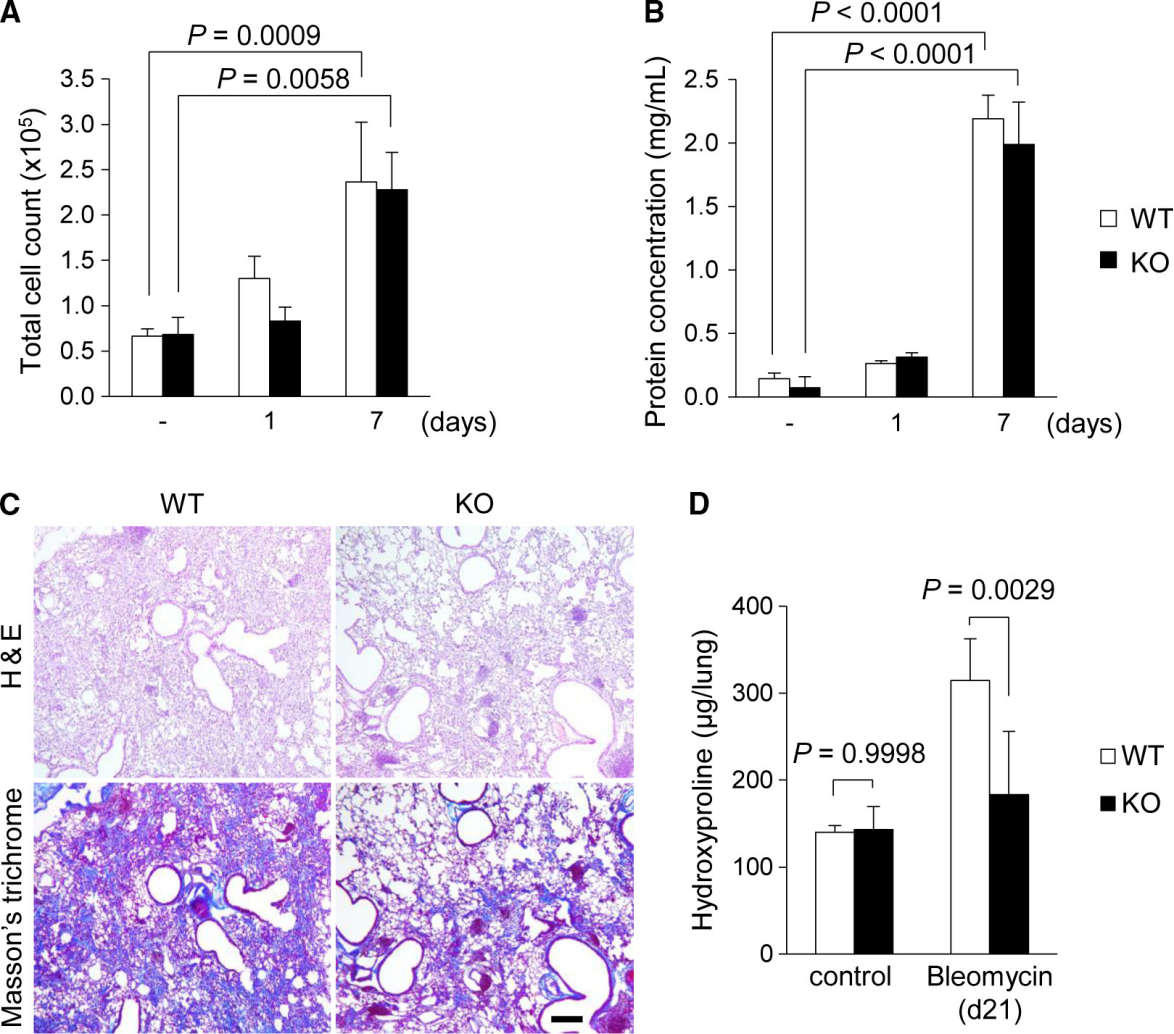
Comparison of inflammatory and fibrotic responses in the lungs between WT and LRG KO mice after bleomycin administration. (A and B) Total cell counts (A) and concentration of total protein (B) in BALF collected from WT and KO mice with or without bleomycin treatment (*n* = 3–5 per group). Mice were sacrificed on day 1 and day 7 after bleomycin treatment for BALF collection. (C) H&E staining and Masson's trichrome staining of the lungs of WT and KO mice 21 days after bleomycin administration. Scale bar = 100 *μ*m. (D) Hydroxyproline contents of the whole lung of WT and KO mice measured by colorimetric method (*n* = 3–7 per group). One way ANOVA followed by Tukey's test was used for statistical analysis.

To investigate the role of LRG in lung fibrosis, LRG KO mice were treated with bleomycin. The total cell count (Fig. 2A) and protein concentration (Fig. 2B) in BALF were significantly increased on day 7 after bleomycin administration in both WT and LRG KO, which suggested that the initial inflammatory responses in the lung did not differ between groups. Next, we evaluated fibrotic response in the lung at day 21 by histological analyses (Fig. 2C). The lung section from bleomycin‐administered WT mice showed distinct fibrosis associated with cell infiltration into alveolar spaces and increased formation of extracellular matrix, whereas in the lungs from LRG KO mice, fibrotic changes were less severe than those from WT mice. In accordance with this observation, the fibrotic area visualized as blue by Masson's trichrome staining was less marked in KO mouse lung than in WT mouse lung. Moreover, lung hydroxyproline content quantified by Ehrlich's reaction was significantly lower in KO mice than in WT mice (Fig. 2D). These observations indicate that the fibrotic lesion consisting mainly of collagen fiber was less severe in LRG KO mice than in WT mice.

### Phosphorylation of Smad2 in the bleomycin‐treated lung was impaired in LRG KO mice

**Figure 3:**
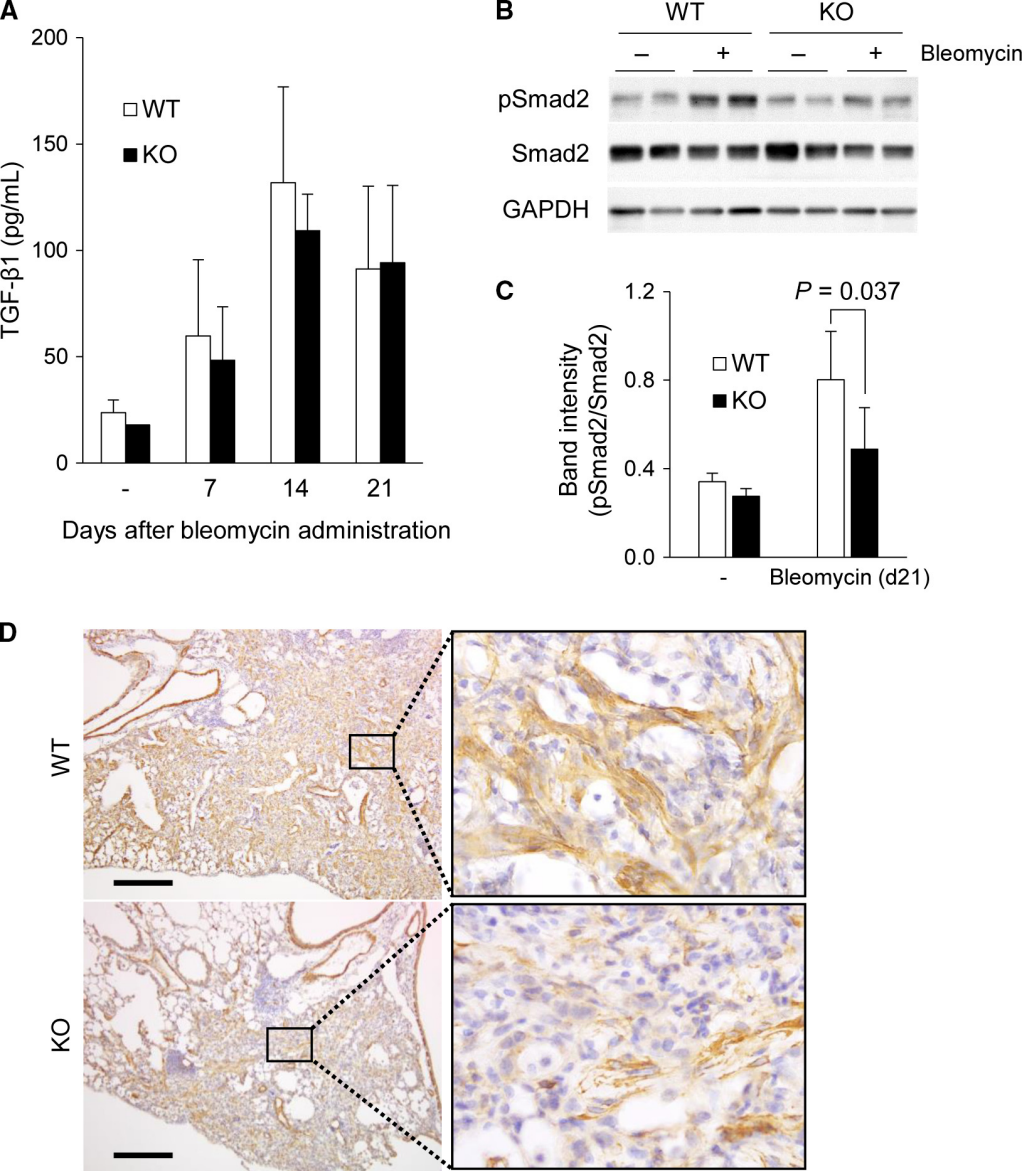
Concentration of TGF‐*β*1 in BALF and activation of Smad2 signaling in lungs from WT and LRG KO mice after bleomycin administration. (A) Comparison of TGF‐*β* levels in BALF between WT and LRG KO mice untreated (‐), and 7, 14 and 21 days after administration measured by ELISA (*n* = 3–5 per group). (B) Phospho‐Smad2 and Smad2 levels in the lungs of WT mice and KO mice 21 days after bleomycin administration detected by western blot analysis. GAPDH was used as an internal control. (C) Quantification of pSmad2 band intensity normalized to Smad2 (*n* = 5). Data were shown as mean ± SD. (D) Immunohistochemical analysis of *α*‐SMA in the lungs of WT and KO mice 21 days after administration. Scale bar = 200 *μ*m.

To elucidate the effect of LRG on TGF‐*β*‐Smad2 signaling in lung fibrosis, we investigated whether the absence of LRG affects TGF‐*β* production and/or signaling. First, we compared the amount of TGF‐*β*1 in BALF between WT and KO mice during fibrosis. The concentration of TGF‐*β*1 was increased at day 7 after bleomycin administration and reached the maximum at day 14 when the first sign of fibrosis was observed. In accordance with the identical inflammatory response between WT mice and KO mice (Fig. 2A and B), no significant difference in TGF‐*β*1 levels was observed at each time point between KO mice and WT mice (Fig. 3A). We therefore considered that the suppressed fibrosis in KO mice may be due to the attenuation of TGF‐*β* signaling, and we investigated the phosphorylation of Smad2 in the lung at day 21 using western blot analysis (Fig. 3B). The signal intensity of phosphorylated Smad2, normalized to Smad2 by densitometric analysis in KO mouse lung was significantly weaker than that in WT mouse lung (Fig. 3C). We then examined the expression of *α*‐SMA, a cytoskeletal marker of myofibroblasts, in lung lesions 21 days after bleomycin administration by immunostaining. In WT mouse lung, *α*‐SMA‐positive fibroblast‐like cells were accumulated at the fibrotic area, whereas in KO mouse lung, accumulation of these cells was not as marked as that in WT mouse lung (Fig. 3D). Myofibroblasts have an important role in the pathogenesis of pulmonary fibrosis by producing collagen and by expressing contractile phenotype, which is controlled by TGF‐*β*‐induced Smad signaling (Zhang et al. 1996; Cao et al. 2010). These results suggest that the TGF‐*β*‐Smad2 pathway in the lung was impaired and the generation of *α*‐SMA‐positive myofibroblasts was attenuated in the absence of LRG.

### LRG enhanced TGF‐*β*‐Smad2 signaling in fibroblasts

**Figure 4:**
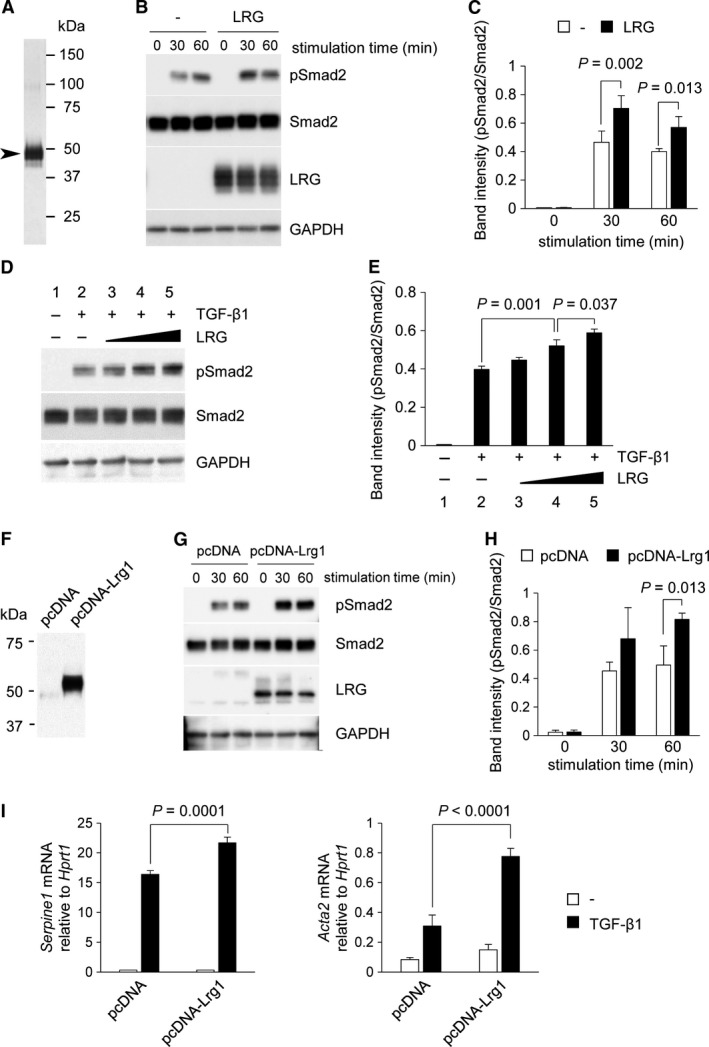
Effect of LRG on TGF‐*β*‐induced Smad2 phosphorylation and downstream gene expression in L929. (A) Silver staining of affinity‐purified recombinant mouse LRG obtained from the culture supernatant of recombinant mouse LRG‐expressing A549 cells using an anti‐LRG antibody‐conjugated column. The sizes of molecular weight markers are indicated on the right lane. The size of the major band (indicated by arrowhead) corresponded to that of LRG (45–50 kDa). (B and C) Detection of phospho‐Smad2 by western blot analysis. L929 cells were treated with or without 20 *μ*g/mL of LRG for 24 h. Cells were then stimulated with 2 ng/mL of TGF‐*β*1 for 0, 30, and 60 min. Anti‐phospho‐Smad2 (Ser465/467), anti‐Smad2, anti‐LRG, and anti‐GAPDH antibodies were used for the detection. Band intensity of pSmad2 normalized to Smad2 is shown in Figure 4C (*n* = 3). (D and E) Detection of phospho‐Smad2 by western blot analysis. L929 cells were treated without (lanes 1 and 2) or with 0.035 *μ*g/mL (lane 3), 0.35 *μ*g/mL (lane 4), and 3.5 *μ*g/mL (lane 5) of LRG for 24 h and then with 2 ng/mL of TGF‐*β*1 for 30 min. Band intensity of pSmad2 normalized to Smad2 is shown in Figure 4E (*n* = 3). (F) Detection of LRG in culture supernatants of pcDNA‐Lrg1 (containing full length LRG cDNA) but not in those of pcDNA (vector without insertion). Supernatants from L929 cells after 24‐h culture in serum‐starved media were analyzed by western blot. (G and H) Detection of phospho‐Smad2 in pcDNA‐Lrg1 and pcDNA cells after treatment with 2 ng/mL of TGF‐*β*1 for 0, 30, and 60 min. Band intensity of pSmad2 normalized to Smad2 is shown in Figure 4H (*n* = 3). (I) qPCR analysis of *Serpine1* and *Acta2*. L929 cells were treated with 2 ng/mL of TGF‐*β*1 for 6 h for *Serpine1* or for 12 h for *Acta2*. The relative expressions of *Serpine1* and *Acta2*, normalized to *Hprt1* gene expression are shown.

Our study of the lung fibrosis model suggests that LRG contributes to the activation of Smad2 signaling in the fibrotic lung. To perform a detailed analysis of the molecular mechanism of LRG in fibrosis, we used fibroblast cell lines, a key cell type for TGF‐*β*‐induced ECM production and fibrosis. To investigate whether LRG directly enhances TGF‐*β*‐induced Smad2 phosphorylation in fibroblasts, affinity‐purified recombinant LRG was prepared (Fig. 4A, see Materials and Methods) and was added to the culture media of L929 mouse fibroblasts that do not express LRG. Western blot analysis showed that LRG enhanced the phosphorylation of Smad2 in TGF‐*β*1‐treated L929 (Fig. 4B). The band intensity of phospho‐Smad2 normalized to total Smad2 was significantly increased by LRG (Fig. 4C) in a dose‐dependent manner (Fig. 4D and E). To exclude the possibility that this reaction was caused by invisible contaminants carried over during the purification process, we generated a stable Lrg1‐expressing cell line (pcDNA‐Lrg1) and control cell line (pcDNA) that was transfected with empty vector only. LRG was detected in the culture supernatants (Fig. 4F) and cell lysates (Fig. 4G, third panel from the top) from pcDNA‐Lrg1 cells but not from pcDNA cells using western blot analysis. Then, we treated these cells with TGF‐*β*1 and evaluated Smad2 phosphorylation. The intensity of phospho‐Smad2 signals after TGF‐*β*1 stimulation was stronger in pcDNA‐Lrg1 cells than in pcDNA cells (Fig. 4G and H). These results strongly suggest the involvement of LRG itself in enhanced Smad2 phosphorylation. We next analyzed the expression of genes downstream of Smad2 by quantitative PCR. As shown in Figure 4F, the expression levels of *Serpine1* and *Acta2* in response to TGF‐*β*1 stimulation were significantly higher in pcDNA‐Lrg1 cells than in pcDNA cells. Thus, our in vitro study indicates that LRG enhances TGF‐*β*‐induced Smad2 phosphorylation in fibroblasts and thereby contributes to development of fibrosis.

### Endoglin was not required for the effect of LRG on TGF‐*β*‐Smad2 signaling in fibroblasts

**Figure 5:**
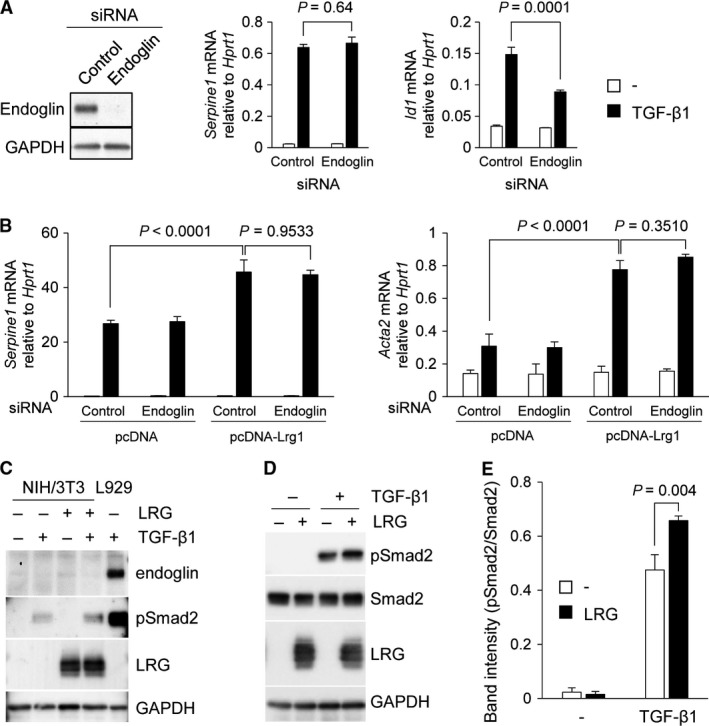
Contribution of endoglin to the effect of LRG on TGF‐*β*‐Smad2 signaling in fibroblasts. (A) (Left) Detection of endoglin in L929 cells by western blot analysis. Cells were transfected with endoglin siRNA or control siRNA and incubated for 24 h. GAPDH was used as an internal control. (Middle and right) qPCR analysis of *Serpine1* and *Id1*. L929 cells were treated with 2 ng/mL of TGF‐*β*1 for 6 h for *Serpine1* or for 1 h for *Id1*. The relative expressions of *Serpine1* and *Id1*, normalized to *Hprt1* gene expression are shown. (B) qPCR analysis of *Serpine1* and *Acta2*. L929 cells were transfected with endoglin siRNA or control siRNA and then treated with 2 ng/mL of TGF‐*β*1 for 6 h for *Serpine1* or 12 h for *Acta2*. The relative expressions of *Serpine1* and *Acta2*, normalized to *Hprt1* gene expression are shown. Two‐way ANOVA followed by Tukey's test was used for statistical analysis. (C) Detection of endoglin in NIH/3T3 cells by western blot analysis. Cells were treated with 2 ng/mL of TGF‐*β* and/or 20 *μ*g/mL of LRG for 24 h. L929 treated with 2 ng/mL of TGF‐*β* for 60 min was used as a positive control for endoglin and pSmad2. (D and E) Detection of phospho‐Smad2 in parental NIH/3T3 cells. Cells were treated with 20 *μ*g/mL of affinity‐purified LRG for 24 h and then treated with 2 ng/mL of TGF‐*β*1 for 30 min. Phospho‐Smad2, Smad2, LRG, and GAPDH were detected by western blot analysis. Band intensity of pSmad2 normalized to Smad2 is shown in Figure 5E (*n* = 3).

To determine if endoglin, which has been reported to interact with LRG in endothelial cells, contributes to the effect of LRG on TGF‐*β*‐Smad2 signaling in L929 cells, we first performed a knockdown experiment using endoglin siRNA. Expression of endoglin in L929 was suppressed by siRNA transfection, as determined by western blot analysis (Fig. 5A, left). The effect of endoglin knockdown was also checked by suppression of *Id1* gene induction, which is dependent on endoglin (Fig. 5A, middle and right). Control siRNA treatment did not affect induction of the *Serpine1* gene in TGF‐*β*‐treated LRG‐expressing cells, as shown in Figure 5B. Interestingly, the *Serpine1* induction remained high in pcDNA‐Lrg1 cells even when endoglin expression was suppressed by siRNA (Fig. 5B). An identical result was obtained for the expression level of *Acta2*. These results showed that the enhancement of *Serpine1* and *Acta2* gene expressions by LRG was independent of endoglin in L929 cells.

To confirm the effect of LRG on TGF‐*β*‐Smad2 signaling in a different fibroblast cell line, we used NIH/3T3 cells that do not express endogenous endoglin (Pericacho et al. 2013). We confirmed that neither TGF‐*β*1 nor LRG treatment induced the expression of endoglin in NIH/3T3 cells (Fig. 5C). Smad2 phosphorylation in TGF‐*β*1‐treated NIH/3T3 cells was enhanced by LRG, which corresponded to our observation in L929 cells (Fig. 5D and E). These results suggest that endoglin expression is not required for the process of enhancing TGF‐*β*‐induced Smad2 signaling by LRG in fibroblasts.

### LRG did not enhance TGF‐*β*‐Smad1/5/8 signaling in fibroblasts in the presence of endoglin

**Figure 6:**
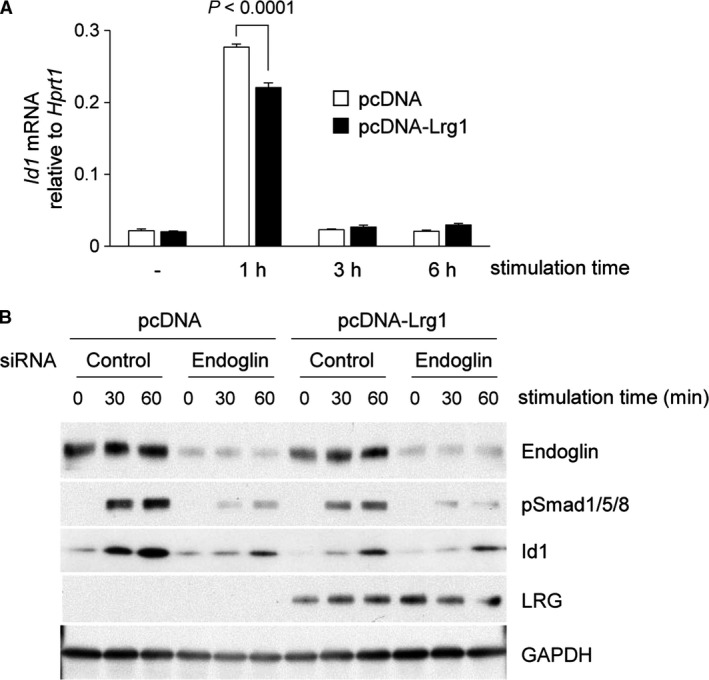
Effect of LRG on TGF‐*β*‐induced Smad1/5/8 phosphorylation and downstream gene expression in L929. (A) qPCR analysis of *Id1*. L929 cells were treated with 2 ng/mL of TGF‐*β*1 for 1, 3, and 6 h. The relative expression of *Id1*, normalized to *Hprt1* gene expression is shown. (B) Detection of phospho‐Smad1/5/8 and Id1 by western blot analysis. L929 cells were transfected with endoglin siRNA and control siRNA and then treated with 2 ng/mL of TGF‐*β*1 for 0, 30, and 60 min. Endoglin, phospho‐Smad1/5 (Ser463/465)/8 (Ser426/428), Id1, LRG, and GAPDH were detected by western blot analysis.

Consideration should be given to TGF‐*β*‐Smad1/5/8 signaling in L929 cells because LRG has been reported to modulate Smad1/5/8 signaling in endothelial cells. Interestingly, *Id1* expression, downstream of Smad1/5/8, was lower in TGF‐*β*‐treated pcDNA‐Lrg1 cells than in pcDNA cells (*P* < 0.0001 at 1 h, Fig. 6A). When cells were treated with endoglin siRNA, Smad1/5/8 phosphorylation and Id1 expression were notably suppressed both in pcDNA‐Lrg1 and pcDNA cells (Fig. 6B). These results suggest that in the presence of endoglin, LRG does not enhance Smad1/5/8 signaling in fibroblasts but instead appears to inhibit it.

## Discussion

IPF is a chronic irreversible disease characterized by progressive decline in lung function and poor prognosis (Raghu et al. 2011, 2015). The participation of TGF‐*β* in the pathogenesis of IPF has been well described, and targeting TGF‐*β* is considered to be a promising approach to treatment (Fernandez and Eickelberg 2012). Therefore, we investigated the involvement of LRG, a molecule that was recently reported to be a modulator of TGF‐*β* signaling (Wang et al. 2013; Takemoto et al. 2015) in the pathogenesis of the fibrotic disease. In this study, we demonstrated that LRG was upregulated in the bleomycin‐treated WT lung, and lung fibrosis was less severe in the absence of LRG. In addition, Smad2 phosphorylation, downstream of TGF‐*β* signaling was attenuated in the lungs of LRG KO mice relative to that in the lungs of WT mice. Furthermore, LRG enhanced TGF‐*β*‐induced Smad2 signaling in a cultured fibroblast cell line. These results suggest that LRG acts on fibroblasts to enhance collagen production in the lung *via* TGF‐*β*‐induced Smad2 activation. The finding that LRG is involved in lung fibrosis by functioning as an enhancer of TGF‐*β* signaling provides a new potential approach to treat IPF by targeting TGF‐*β*‐associated protein.

One potential molecule that determines which cascade is modulated by LRG is endoglin, an accessory TGF‐*β* receptor. It has been discussed that the lack of endoglin expression in LLC and Hep3B cells results in promotion of the TGF‐*β*‐Smad2 cascade instead of the Smad1/5 cascade by LRG (Takemoto et al. 2015). However, in this study, endoglin was not involved in the process of enhancing TGF‐*β*‐induced Smad2 phosphorylation and expression of fibrosis‐related genes in L929 cells (Fig. 5B). Furthermore, we found that TGF‐*β*‐induced Smad2 phosphorylation was promoted by addition of LRG in NIH/3T3 cells that do not express endoglin (Fig. 5D and E). Our observations strongly suggest that endoglin is not necessary for LRG to enhance Smad2 activation in myofibroblast differentiation.

Endoglin, originally identified as a marker of endothelial cells, is expressed in a variety of cell types, including endothelial cells, monocytes, macrophages, and fibroblasts. Several studies have demonstrated upregulation of endoglin in fibroblasts in fibrotic processes, such as in cutaneous fibroblasts from systemic sclerosis and intestinal fibroblasts from Crohn's disease with strictures (Leask et al. 2002; Burke et al. 2010), whereas endoglin expression in lung fibroblasts has not been well described. Immunohistochemical staining of bleomycin‐treated lung indicated that expression of endoglin in fibroblasts in fibrotic lesions was low relative to that in vascular endothelial cells (data not shown). Our in vitro experiment showed that TGF‐*β*‐induced Smad1/5/8 activation was markedly suppressed in L929 cells treated with endoglin siRNA (Fig. 6B). In addition, in NIH/3T3 cells that do not express endogenous endoglin, Smad1/5/8 activation after TGF‐*β* stimulation was much weaker than the strong signal of phospho‐Smad2 (data not shown). These results led us to speculate that LRG promotes fibrosis mainly by enhancing TGF‐*β*‐induced, endoglin‐independent Smad2 signaling.

Contradictory to a previous study in which LRG promoted Smad1/5/8 signaling in endothelial cells, LRG did not enhance Smad1/5/8 signaling in fibroblasts, but rather seemed to inhibit it (Fig. 6). One possible mechanism to explain this discrepancy is that endoglin has two isoforms produced by alternative splicing called L (long)‐endoglin and S (short)‐endoglin and the different expression patterns of endoglin lead to conflicting results regarding Smad1/5/8. It is known that endothelial cells express both L‐ and S‐endoglin, whereas L929 cells express only L‐endoglin (Perez‐Gomez et al. 2005), which led us to assume that S‐endoglin, a specific isoform for endothelial cells, is essential for LRG to enhance Smad1/5/8, and the effect is abolished or even reversed in the absence of S‐endoglin. Considering that S‐ and L‐endoglin have a common extracellular domain and presumably interact with LRG (Perez‐Gomez et al. 2005; Wang et al. 2013), intracellular events downstream of the TGF‐*β* receptor complex involving endoglin may modulate the effect of LRG on Smad1/5/8 signaling.

The involvement of the Smad1/5/8 signaling pathway during fibrosis has been controversial. In the fibroblasts of patients with systemic sclerosis, the expression of the phospho‐Smad1 protein is increased relative to fibroblasts from normal control biopsies, accompanied by increased expression of type I collagen (Morris et al. 2011). On the other hand, increased Smad1/5 phosphorylation and the upregulation of *Id1,* downstream of Smad1/5, has been shown to result in the inhibition of profibrotic responses in fibroblasts (Cao et al. 2010). A previous study using Id1 knockout mice has indicated that Id1 has an antifibrotic effect in the lungs and inhibits myofibroblast differentiation (Lin et al. 2008). The attenuated TGF‐*β*‐induced Smad1/5/8 activation in LRG‐expressing cells (Fig. 6) may also be involved in the promotion of fibrosis.

An immunohistochemical observation of LRG expression in epithelial and endothelial cells led us to speculate that LRG also plays a role in these cells. A recent study has demonstrated that LRG can enhance epithelial–mesenchymal transition (EMT) in human colorectal cancer cells (Zhang et al. 2016). Moreover, the effect of LRG on endothelial cells has been documented in pathogenic retinal neovascularization (Wang et al. 2013). Studies using animal models have demonstrated that EMT (Kasai et al. 2005; Hinz et al. 2007) and aberrant angiogenesis (Wan et al. 2013) are important processes in the development of lung fibrosis. We can surmise that there exist multiple mechanisms underlying the effects of LRG in pulmonary fibrosis.

Our observations here raise hope that LRG is a potential therapeutic target for diseases with tissue fibrosis. In a study involving a proteomic approach to identify blood biomarkers in IPF, LRG was found in the list of differentially expressed proteins that are upregulated in patients with IPF compared with healthy subjects (Niu et al. 2017). In our in vitro study, serum LRG showed a slight increase in bleomycin‐treated mice compared with control mice (Fig. 1C). This leads us to suppose that LRG in the lung lesions in patients with IPF may be upregulated, as we observed in the mouse model of pulmonary fibrosis. Fortunately, LRG KO mice have no serious abnormalities in growth and reproduction, unlike other TGF‐*β*‐related gene knockout mice, such as endoglin and ALK1 (Li et al. 1999; Oh et al. 2000). The inhibition of LRG may provide an effective treatment for fibrotic diseases without having serious adverse effects related to homeostatic and physiological functions of TGF‐*β*.

In conclusion, our study demonstrated that LRG enhanced TGF‐*β*‐Smad2 signaling in fibroblasts and participated in fibrotic processes in vivo and in vitro. Our findings provide new insights into therapeutic strategies for fibrotic lung diseases, including IPF.

## Conflict of Interest

T. Naka is receiving a grant from SEKISUI MEDICAL CO., LTD. For the remaining authors, there are no conflicts of interest or financial interests.
